# A Study of Some of the Causes of Failure in Dental Operations—A Plea for More Scientific Professional Methods

**Published:** 1893-01

**Authors:** L. Ashley Faught

**Affiliations:** Philadelphia


### A STUDY OF SOME OF THE CAUSES OF FAILURE IN DENTAL OPERATIONS—A PLEA FOR MORE SCIENTIFIC PROFESSIONAL METHODS.[1]

BY L. ASHLEY FAUGHT, D.D.S., PHILADELPHIA.

Mr. President and Gentlemen,—It is my privilege and pleasure to address you to-night upon what has been to me a cherished hope; and I earnestly desire to bring to you a message which shall impel the adoption of such methods of practice as will make that hope a living fact. I do not look for any immediate result, but I believe that concerted action relative to them now will not be without its impress upon the possibilities of the future.

On the 21st day of April, 1885,—a few days more than just seven years ago,—in a paper read before the Odontographic Society of Pennsylvania,[2] I placed before the profession "The Legal Standing of Dentistry," and have since labored incessantly to secure to it that status. Others, too, have entered most heartily upon the work, and not a little of what was then advocated has been accomplished; but the real and vital point is still unattained. A true, separate, legal standing as a profession does not exist. We can but pretend to-day to an advancement towards it, and this advancement is to be helped by the use of more scientific professional methods.

You will, therefore, perceive that I desire not only to interest you in a study of some of the causes of failure in dental operations, but that I also mainly wish that interest to lead to a general adoption of more scientific professional methods, to the end that the legal standing of dentistry may be secured.

That I may not be accused of making mere assertion of the premise,—no legal standing,—I may be allowed to tersely recall to your minds and to my own the outlines of the position.

The relative aspects of dentistry are, the relation of dentists to dentistry, the relation of the public to dentistry, the relation of physicians to dentistry, and the relation of the law to dentistry.

The relation of dentists to dentistry is inexact, for each individual practitioner, according to the amount of culture and education possessed, and the circumstances surrounding him in practice, grades it differently,—all the way from what is high and comprehensive to

---

[1] Read before the Central Dental Association of Northern New Jersey, May 16, 1892.

[2] See the *Dental Practitioner*, vol. iii., No. 6, p. 132.

what is low and narrow,—expressing in teaching, in literature, in scientific society contact, both a manual and medical aspect, but carrying into every-day practice little else than routine manipulation. True dentistry, in the highest and only proper sense of that word, is to-day the broadest single branch of the healing art, and is capable of giving to the public a service as good and as valuable as is received in response to the majority of demands made upon physicians; but the public do not think of the services of the dentist in parallel lines with the services of the physician, either in appreciation or in the monetary acknowledgment they are willing to render for the same. They seldom approach him with other ideas of the scope of his powers than that he has the simple ability to fill a tooth, to extract a tooth, and to insert an artificial denture.

I do not propose, in speaking of the relation of physicians to dentistry, to revive here for one moment what I broadly discussed seven years ago. The years that have elapsed since then have been busy years, replete with many discussions and many conflicts of opinion, and the work has been thoroughly done, for we now propose to have a World's Columbian Dental Congress in 1893. No intelligent physician (M.D.) or dentist (D.D.S.) to-day thinks of dentistry as other than a profession. Each sees in his own domain an illimitable field of pathological study, sufficient for his desires and more than he can deal correctly with in a lifetime. The physician in particular knows that he peculiarly knows nothing of dentistry. That hazy blending of dentistry as a specialty in medicine is at last recognized to be a fiction indigenous to the minds of hybrids (M.D., D.D.S.).

The line of thought through which I have led you this evening shows dentistry to be in an anomalous position. Meaning as it does the care of the teeth, of the near and remote lesions having an influence on those organs, and of the medical and surgical aspects influencing them, it is recognized by physicians, who know that they understand but little, and that little indefinitely about it, to be in the hands of a class of men abundantly able to cope with every difficulty, and who are consecrated to a high professional attainment; but, nevertheless, it is so placed that we are unable to deal with a case eventuating in death (rare though it may be) with dignity and safety to ourselves and to our profession. Until the law, which causes dentists to qualify under a distinct degree, perfectly protects them in this direction, dentistry is without legal standing, and I fear that it will continue to give in large measure, and the public to expect, only a manual practice. May our study to-night lead to a more general adoption of more scientific methods of practice, and bring forth more scientific service, that these may help to secure for us a legal standing.

Recognizing that the thoughtful student always learns more by his failures than by his successes, I have chosen that we should consider the causes of failure in dental operations. Who is there who does not have failures? Who is there here to-night who has not time and again been humbled just as he was about to rejoice in the pride of his strength, and made to realize that he was not yet the perfect master of all things? In careers filled with successes we meet with failures, and a sufficient answer is constantly sought to the query, Wherefore?

Accepted methods of practice suggest that "failure in dental operations is mainly due to defective manipulation."

New departure practice says, "Failure of dental operations is mainly due to incompatibility of filling-material with tooth-bone."

And in 1887 your essayist offered the following sentiment: "Failure in operation and tooth-loss are mainly due to the lack of oral hygiene."

Certainly within the compass of these three replies is to be found much in explanation of the failures which occur; but my purpose to-night is to look somewhat back of these and at the more minute things which influence the results of our labors. Let us consider a few of those most prominent in my experience. In the first place, then, I am convinced that in filling teeth we often fail of success because we are not complete masters of the situation at the time of the operation.

The cavity to be filled should be so protected from dampness that perfect dryness may be assured at each succeeding step. Moisture threatening towards the close of an operation need not actually get into the cavity to influence deleteriously the future result. The hurried and worried efforts to reach completion dry, even though successful, are in themselves almost as sure a factor to produce future failure as would have been the moisture. We should also decline to operate when the space is insufficient through which to do the work. The fact that an appointment has been made, and that we expected, or the patient expected, the operation to be accomplished at that particular time, rarely should be reason sufficient for an attempt to render a service which we recognize as possible to be imperfect under the conditions.

I consider it essential in filling approximal cavities to invariably cut the enamel margins back sufficiently, that we may secure abso- ute metallic contacts only between the facing fillings, or between the filling and the facing tooth, with the line of junction of filling-material and tooth-tissue so exposed as to be capable of thorough cleansing. Other conditions than this are to be considered a frequent cause of failure.

Up to this date proper sterilization of all cavities to be filled seems not to have been sufficiently insisted upon. We live in an age of antiseptics and germicides, and those who ignore their efficacy deprive themselves of agents of the utmost value. Scientific experiments of prominent investigators have shown that momentary application of these agents is but little better than no use of them at all. Proper sterilization of cavities requires the agent to be sealed in the cavity an ascertained length of time. To do this effectively most cavities will require preparation at one sitting and the insertion of the filling at another.

Now, gentlemen, I am well aware that proper space, proper control of conditions of moisture, and proper sterilization entails upon the operator labor for which he should be remunerated, and upon the patient consumption of time, for which he should be willing to give remuneration; and I know, too, that just here is the difficulty. The operator that conscientiously follows such high methods of scientific practice is, under the present status of dentistry, likely to find himself engaged in giving a service for which it will be difficult to obtain proper reward.

That difficulty would in large measure vanish if the profession as a profession would do its duty, and give a service in full accord with its knowledge. It would also lead to what to me is dearer than proper remuneration,—proper recognition in the thoughts of the public that dentistry is scientific in its methods of practice, and capable of something more than the scraping out of a little decay and the stuffing in of a little gold.

There is nothing more to be condemned than the putting on of rubber dam without prior medication around the necks of the teeth and of the adjacent gums upon which it impinges, and yet, collectively, we go on adjusting it day in and day out, smiling as we put it on, and pretending that the pain is but slight, building up in the minds of sensitive, shrinking patients a dread of us and of our methods. They are unscientific; and is it any wonder that we are not more cherished and more loved? That such are the facts of the practice of to-day is not the fault of scientific dentistry; it is the fault of its practitioners. There are means of relief that we know of, that we talk of, but which we do not use. I plead, therefore, to-night for the adoption of more scientific methods of practice. Thrust not the lancet into quivering humanity without proper local anæsthesia, even though each cut is known to be the antiphlogistic touch of a therapeutic knife.

To reconstruct the position of dentistry with the public, we must adopt these scientific methods as a profession. One, two, three, or a dozen gentlemen cannot alone reverse the established title of error.

In my opinion the treatment of acute abscess should be relegated from the office to the patient's house, and regular visitations made by the dentist during the continuance of active inflammation. What science would there be in a physician allowing a patient with racked nerves, elevated temperature, and the whole ensemble of acute illness from abscesses in other portions of the economy to expose himself to the possible contingencies of weather, etc., by making visits to him at his office? Nothing scientific whatever, only malpractice. Is dentistry so empirical as to expect good results from such gross violation of the first principles of healing?

I have long believed that one of the chief causes of failure in dental operations is the fact that the dentist sees only a cavity to be filled, and that he responds to the only demand on the part of the patient, "I want a cavity filled." The plug goes into the cavity, and this is dentistry. More proper would it be for the patient to consult the dentist for professional advice. More proper for the dentist to examine the patient before doing anything or giving that advice. Inquiry into the physical condition of the patient at the time of the proposed operation, study of the temperament, character of tooth-tissue, environment, etc., should be the first essentials of the visits to the dentist, the filling of cavities a sequential matter only, and entirely subsequent. Each operation should be given its proper place in full compatibility with the essentials for success. The more tedious operations at the hours of the day when both operator and patient are at their physical best, and at no other time should they be done. With female patients, knowledge of their periods should be acquired, and dental operations suspended or reduced to a minimum during their continuance. I may be repeating to you here things with which you are all conversant, but it is because I believe the failure of many dental operations is due to the non-application in practice of the scientific knowledge which we have, and I desire to connect in your minds one with the other.

Most patients approach dental operations with dread; almost all suffer a nervous strain from even the slightest service. A better result is possible with a quiet and submissive patient than with a restless one. This apprehensive and nervous condition of the patient increases with the return of each sitting during a line of work. The remembrances of what the patient has passed through during a given service is a deterring factor to a frequent and near visitation in the future for additional service or for examination. The nervous condition of the patient, therefore, is a factor in the results of dental operations, and far more successes and less failures would be met if the dentist would adopt proper medical systemic treatment before and after operations, and peculiarly is this true during the visits of protracted lines of work. Proper sedatives or tonics should be recommended before operation. Proper sedatives or tonics after operations. It may seem strange to you that I should say sedative or tonic, and yet it is true that you must determine in each patient which is indicated. It will not do for you to judge by the knowledge of temperaments which you may possess, or by the statements of the patients as to their nervous condition. You must in each instance scientifically determine it and the nature of it,—that is, whether the nervous anticipation stimulates or depresses the system; whether the nervous condition is anticipatory or most during the operation. For the purpose of determining these conditions, I propose the use of the clinical temperature thermometer. The following record of cases will illustrate my meaning:

| Case. | Sex. | Temperament. | Age. | First temperature. | Second temperature. | Operation. |
|---|---|---|---|---|---|---|
| 1 | Female. | Bilio-nervous. | 40 | $98\frac{3}{5}°$ | $99\frac{2}{5}°$ | Plastic guard. |
| 2 | Male. | Lymphatico-nervous. | 19 | $98\frac{3}{5}°$ | $99\frac{2}{5}°$ | Gold filling. |
| 3 | Female. | Nervous. | 25 | 99° | $99\frac{4}{5}°$ | Alloy plug. |
| 4 | Female. | Nervous. | 21 | $100\frac{1}{5}°$ | $100\frac{3}{5}°$ | Arsenic application. |
| 5 | Female. | Nervo-bilious. | 42 | 99° | 100° | Filling ordinary. |
| 6 | Female. | Nervo-lymphatic. | 30 | $97\frac{2}{5}°$ | $99\frac{2}{5}°$ | Large contour filling, gold. |
| 7 | Female. | Bilious. | 32 | $99\frac{3}{5}°$ | $98\frac{4}{5}°$ | Alloy fillings. |
| 8 | Female. | Bilio-lymphatic. | 30 | $99\frac{2}{5}°$ | $99\frac{2}{5}°$ | Alloy and gold fillings. |
| 9 | Female. | Bilio-nervous. | 30 | $99\frac{3}{5}°$ | $99\frac{1}{5}°$ | Gutta-percha plug. |
| 10 | Female. | Bilio-nervous. | 35 | $98\frac{3}{5}°$ | $98\frac{3}{5}°$ | Gold fillings. |
| 11 | Female. | Lymphatic. | 30 | $98\frac{3}{5}°$ | $98\frac{3}{5}°$ | Gold fillings. |
| 12 | Female. | Nervous. | 25 | $98\frac{2}{5}°$ | $100\frac{1}{5}°$ | Alloy filling; no preparation. |
| 13 | Female. | Bilious. | 21 | $98\frac{3}{5}°$ | $97\frac{2}{5}°$ | Cleaning teeth; not scaling. |
| 14 | Male. | Bilio-lymphatic. | 50 | $98\frac{3}{5}°$ | $99\frac{3}{5}°$ | Arsenic application. |
| 15 | Female. | Nervous. | 36 | $98\frac{3}{5}°$ | $99\frac{3}{5}°$ | Examination. |
| 16 | Female. | Bilio-lymphatic. | 37 | $99\frac{3}{5}°$ | 99° | Examination. |
| 17 | Female. | Nervo-bilious. | 18 | $98\frac{3}{5}°$ | $99\frac{2}{5}°$ | Gutta-percha filling. |
| 18 | Female. | Nervo-lymphatic. | 28 | $98\frac{4}{5}°$ | 99° | Gold filling. |
| 19 | Male. | Bilio-lymphatic. | 40 | $99\frac{4}{5}°$ | $99\frac{3}{5}°$ | Plastic filling. |

In an analysis of a table such as this there is much food for thought and study. A cursory review of it, however, shows that in some patients the elevation of temperature is greater before the operation than during it, that in the majority of cases the rise is during the operation rather than in anticipation of it. The depressed temperature in Cases 6 and 13 is suggestive of a possible condition in which a patient might die under gas extraction and the dentist receive the credit. Certainly those patients at the time were not in condition for the insertion of a large gold plug. The increase of temperature in anticipation, and a relapse towards normal during the operation, would indicate preparative system treatment rather than medication after the operation. But, gentlemen, I fear wearying you. The field of study is new, and there is an opportunity for you all to make records, and a subsequent contribution to dental literature. I hope that many will push the investigation with me, for I know that the more scientific our methods of practice become the sooner will dentistry have a legal standing accorded to it.

